# Artificial Lightweight Aggregates Made from Pozzolanic Material: A Review on the Method, Physical and Mechanical Properties, Thermal and Microstructure

**DOI:** 10.3390/ma15113929

**Published:** 2022-05-31

**Authors:** Dickson Ling Chuan Hao, Rafiza Abd Razak, Marwan Kheimi, Zarina Yahya, Mohd Mustafa Al Bakri Abdullah, Dumitru Doru Burduhos Nergis, Hamzah Fansuri, Ratna Ediati, Rosnita Mohamed, Alida Abdullah

**Affiliations:** 1 Faculty of Civil Engineering Technology, Universiti Malaysia Perlis (UniMAP), Arau 02600, Perlis, Malaysia; dlch6179@gmail.com (D.L.C.H.); zarinayahya@unimap.edu.my (Z.Y.); 2 Centre of Excellence Geopolymer and Green Technology (CEGeoGTech), Universiti Malaysia Perlis (UniMAP), Arau 02600, Perlis, Malaysia; mustafa_albakri@unimap.edu.my (M.M.A.B.A.); rosnitamohamed21@gmail.com (R.M.); alida@unimap.edu.my (A.A.); 3 Department of Civil Engineering, Faculty of Engineering—Rabigh Branch, King Abdulaziz University, Jeddah 21589, Saudi Arabia; mmkheimi@kau.edu.sa; 4 Faculty of Chemical Engineering Technology, Universiti Malaysia Perlis (UniMAP), Arau 02600, Perlis, Malaysia; 5 Faculty of Materials Science and Engineering, Gheorghe Asachi Technical University of Iasi, 700050 Iasi, Romania; 6 Department of Chemistry, Institut Teknologi Sepuluh Nopember, Surabaya 60115, Indonesia; h.fansuri@chem.its.ac.id (H.F.); rediati@chem.its.ac.id (R.E.)

**Keywords:** artificial lightweight aggregate, geopolymer, aggregate crushing value, aggregate impact value, thermal properties, pozzolanic materials

## Abstract

As the demand for nonrenewable natural resources, such as aggregate, is increasing worldwide, new production of artificial aggregate should be developed. Artificial lightweight aggregate can bring advantages to the construction field due to its lower density, thus reducing the dead load applied to the structural elements. In addition, application of artificial lightweight aggregate in lightweight concrete will produce lower thermal conductivity. However, the production of artificial lightweight aggregate is still limited. Production of artificial lightweight aggregate incorporating waste materials or pozzolanic materials is advantageous and beneficial in terms of being environmentally friendly, as well as lowering carbon dioxide emissions. Moreover, additives, such as geopolymer, have been introduced as one of the alternative construction materials that have been proven to have excellent properties. Thus, this paper will review the production of artificial lightweight aggregate through various methods, including sintering, cold bonding, and autoclaving. The significant properties of artificial lightweight aggregate, including physical and mechanical properties, such as water absorption, crushing strength, and impact value, are reviewed. The properties of concrete, including thermal properties, that utilized artificial lightweight aggregate were also briefly reviewed to highlight the advantages of artificial lightweight aggregate.

## 1. Introduction

Aggregates are widely used in the field of construction, specifically in the manufacture of concrete. The increasing demand for concrete will result in an increase in the requirements of all concrete ingredients, including aggregates, which represent 60–70% of the total volume of the concrete [1]. Most of the aggregates used are obtained from natural resources, including rocks. The concrete industry has an impact on the global environmental problem due to the utilization of large amounts of natural resources. Natural resources are decreasing at a quicker rate because of the high demand for usage in the manufacturing of concrete. The development of lightweight materials, such as lightweight aggregate, will help to minimize the use of natural resources.

In the construction field, lightweight aggregate that is used in concrete production is a type of material that is very environmentally friendly. Lightweight aggregate is dramatically different from conventional aggregate. The modifications bring advantages for the designers for a number of reasons other than weight reduction, such as decreased early cracking, decreased permeability, and longer lifespan [2]. Lightweight aggregates can be grouped into the following categories [3]:

1. Materials that naturally occur and require further processing, such as expanded clay, shale and slate, and vermiculite;
2. Industrial waste by-products, such as sintered pulverized fuel ash (fly ash), foamed or expanded-blast-furnace slag, and hemalite;
3. Materials that naturally occur, such as pumice, foamed lava, volcanic tuff, and porous limestone.

Sintered fly ash aggregate, also known as Lytag, is the most widely used artificial aggregate in the construction field [4]. According to Nadesan and Dinakar [5], sintered fly ash aggregate is capable of producing concrete with high strength performance. In addition, the application of lightweight aggregate in concrete has increased in popularity due to its low density, good thermal conductivity, being environmentally friendly, and many economic advantages [6]. Concrete with lightweight aggregate also has low thermal conductivity [6]. The concrete with lightweight aggregate had lower thermal conductivity [2]. The objective of this paper is to review the manufacturing method of lightweight aggregate for the production of lightweight concrete, as well as the properties of lightweight aggregate. The properties include physical properties, such as specific gravity and water absorption, and mechanical properties, such as crushing strength and aggregate impact value. The mechanical and thermal properties of concrete consisting of lightweight aggregate that have been reported previously are also reported in this review.

## 2. Aggregate

The main component of concrete is aggregates, which occupy around 70% to 80% of the total volume, with fine aggregate accounting for 25% to 30% and coarse aggregate accounting for 40% to 50% [7]. The coarse aggregate that is usually utilized in construction work comes from various types of resources, such as rock, crushed stone, and gravel [8]. Crushed rocks are commonly used as coarse aggregate and river sand as fine aggregate, both of which can be found naturally. A large amount of natural aggregate, such as sand, gravel, or crushed rock, is mined for concrete manufacturing, and the world’s aggregate usage is estimated to be in excess of 40 billion tonnes per year, with concrete accounting for 64% to 75% of all mined aggregate [9]. The massive use of raw materials for aggregates is expected to reach 62.9 billion metric tonnes per year by 2024 with global construction aggregates consumption, and this can deplete natural resources, as it creates an immediate risk to the environment [10]. The construction projects need a considerable amount of natural aggregate for the production of concrete, which increases the depletion of natural aggregate resources and makes the sustainability of the construction projects more challenging [11]. Due to the increasing expansion of building construction, natural aggregate supplies are rapidly decreasing, resulting in a shortage of resources. This shortage of resources requires proper utilization for sustainable growth [7]. The natural sand can be replaced by using by-products of coarse aggregate, such as copper mine waste rocks, as it reduces the manufacturing cost, reduces CO_2_ emissions from the industrial process of producing natural sand, and water consumption for sand washing would be minimized [12]. Manufactured sands are essentially a waste product from the production of coarse aggregate, which are generally available, have a cheaper cost, and would reduce natural sand mining [13]. In addition, manufactured sand had become a popular choice to be used to replace fine aggregate, as it is generally mined from stream beds, and harvesting sand is thought to be environmentally detrimental [14]. In addition, manufactured sand which is made from hard granite rocks can be produced locally by lowering the expense of shipping from a distant river sand bank, and it is dust-free, which is readily regulated to suit the needed grading for the construction [15]. Furthermore, the application of lightweight aggregate in concrete has been developed in order to resolve the depletion of natural aggregate. Due to its advantages in decreasing load bearing and also improving performance in thermal insulation, the development of lightweight concrete by including lightweight aggregates has caught researchers’ attention [16].

## 3. Lightweight Aggregate

A lightweight aggregate (LWA) is a solid substance having a particle density of less than 2.0 g/cm^3^ and a loose bulk density of less than 1.2 g/cm^3^ (BS EN 13055-1, 2002) [17]. LWAs are porous and granular materials that have been widely used in architecture, landscaping, and geotechnics [18]. In addition, it can provide better sound absorption and thermal insulation [9]. Lightweight aggregates are ecologically friendly construction materials made from a variety of wastes and frequently produced through high-temperature burning [19]. There are two types of lightweight aggregate, which are natural lightweight aggregate and artificial lightweight aggregate. The following are the two types of lightweight aggregate:

1. Aggregates that occur naturally and can only be used after mechanical treatment, such as pumice and scoria aggregates [20].
2. Artificial aggregates are made up of waste materials, such as fly ash, husks, or volcanic form and ground granulated blast-furnace slag (GGBS) [21]

Various types of lightweight aggregate have been widely utilized as construction materials in the construction field. Considering aggregate makes up approximately 70% of the concrete mixture, substituting natural aggregate with lightweight aggregate manufactured from waste materials will be an efficient method to minimize nonrenewable resource usage [22]. Lightweight aggregate has been discovered as significant in the formation of lightweight concrete by lowering greenhouse emissions in buildings and decreasing the self-weight of the structure [23]. Application of LWA in concrete will enhance thermal insulation characteristics, decrease structural dead load, allowing larger structures to be built with the same foundation size, and lead to lower CO_2_ emissions [24]. Furthermore, LWA is a critical component in the construction of earthquake-resistant buildings [25]. Due to the larger amount of internal pores in lightweight aggregate, absorption of moisture from cement paste is more rapid than in normal-weight aggregate, which makes the concrete less workable and lower in strength performance than the concrete prepared with normal aggregate [26]. Regardless of the performance when compared to natural aggregate concrete, LWA is worthwhile to be explored specifically for enhancing the performance towards minimizing environmental problems, alongside maintaining long-term sustainability by improving water quality or as a growth medium for green roofs to mitigate the urban heat island effect [27].

### Lightweight Aggregate with Inclusion of Geopolymer

The inclusion of geopolymer in lightweight aggregate has become more concerning due to its advantages in improving the properties of lightweight aggregate. The strength of activated fly ash-based artificial lightweight aggregate by inclusion of geopolymer is comparable to that of commercialized expanded clay lightweight aggregate [22]. In addition, the inclusion of geopolymer in lightweight aggregate produced from fluidized bed combustion (FBC) fly ashes and mine tailings showed excellent mechanical properties in mortar and concrete as compared to the application of commercialized aggregate (LECA) [28]. The inclusion of geopolymer in the lightweight aggregate manufactured from recycled silt and palm oil fuel ash meets the demand for high-strength lightweight concrete and can be utilized for lightweight construction or insulating concrete [23]. In addition, the inclusion of geopolymer in lightweight aggregate that is produced from the combination of fly ash and silica fume can be used for heavy-duty floors due to its high strength [29]. Therefore, the inclusion of geopolymer in lightweight aggregate brings advantages in the construction field, especially in the structural components. However, the use of geopolymer in lightweight aggregate is still limited, and more research is needed to identify lightweight aggregate properties.

## 4. Manufacturing of Lightweight Aggregate

**Figure 1:**
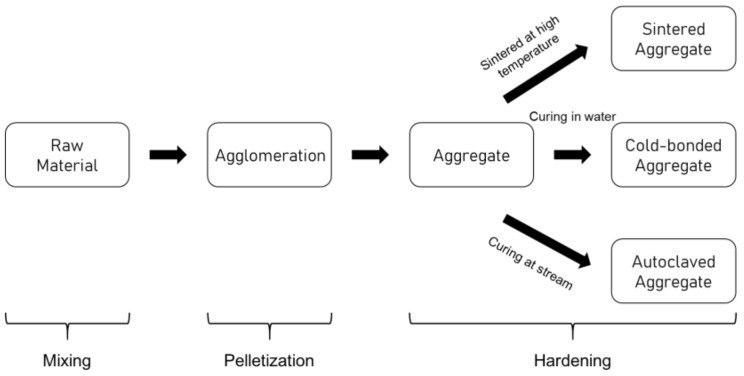
Flow chart of producing lightweight aggregate.

The manufacturing process of artificial aggregate consists of three stages, which are the mixing of raw materials, pelletization, and hardening. In the first stage, which is mixing, the well-proportioned ingredients are mixed until the mixture achieves consistency. In a disc-based pelletizer machine, the mixture of the raw materials undergoes the pelletization process by the agglomeration of the fine particles using a suitable binder. Some previous studies used pozzolanic materials as binders, such as metakaolin and bentonite [5,26,27]. Meanwhile, alkaline activators are commonly used as binders for the production of geopolymer aggregate [18,27,28,29]. Depending on the angle of the disc, the speed of the pelletizer, and the moisture content, the appropriate size of pellets will be collected in the disc. The hardening of the fresh pellets can be accomplished by using sintering, cold bonding, or autoclaving in order to gain the strength of the aggregate. The flow chart of the production of lightweight aggregates can be illustrated as in Figure 1. Meanwhile, the current research on lightweight aggregate is summarized as in Table 1.

In the study of Punlert et al. (2017) [39], lightweight concrete was manufactured using fly ash lightweight aggregates instead of coarse aggregates, resulting in a much lower density and good strength compared to conventional concrete. Furthermore, when sintered at around 1100 °C, lightweight aggregate made from sewage sludge and river sediment achieved high density, low water absorption, and high strength. However, the existence of air voids in fly ash lightweight aggregates, which are crucial for absorbency, leads to difficulty in producing lightweight aggregate concrete, especially in the mix design, which requires further work to enhance the properties [40]. For this purpose, additional binders or additives are introduced as one of the alternatives towards improving the properties of lightweight aggregate.

From the previous results, the salt additives (NaCl) resulted in less viscosity and produced wider internal pores, which allowed the production of ultralight aggregates. However, the use of Na_2_CO_3_ as an additive, which is low cost and low corrosion hazard, allows the creation of ultra-lightweight aggregates [19]. In the study reported by Ren et al. [31], addition of coke particles in the manufacturing of lightweight aggregate will help to reduce the apparent density of the aggregate produced. The use of styrene butadiene rubber (SBR) improves the microstructure of lightweight aggregate, thus improving the aggregate’s mechanical properties [35]. In addition, the inclusion of waste glass powder causes the pozzolanic material to inflate, resulting in a more efficient lightweight aggregate by enhancing the porosity of lightweight aggregate and decreasing water absorption [30].

In addition, pozzolanic materials with high SiO_2_, Al_2_O_3_, and CaO content have a high potential to be utilized in producing artificial aggregates with the addition of an alkaline activator [23]. The usage of alkaline activator as an additive for pozzolanic materials will help to influence the formation of C-S-H binding gel and sodium aluminosilicate hydrates during the geopolymerization process [41]. The formation of geopolymer aggregate by mixing pozzolanic material with alkaline activator will decrease the porosity of aggregate and improve the strength due to the extra C-S-H and calcium reaction during the reaction process alongside the denser microstructure produced [33]. Furthermore, the NaOH molarity will affect the strength of the geopolymer aggregate. For instance, low sodium hydroxide content leads to improper dissolution of fly ash, thus causing the inter-particle spaces of the participating gels to not be entirely filled [35]. It is critical to investigate the optimization of mix designation for each kind of material utilized in the manufacturing of lightweight aggregate-based geopolymer.

To summarize, recent research has shown that fly ash is the common material that had been chosen to produce the lightweight aggregate due to their excellent properties. In the future, alternative pozzolanic materials should be studied to establish their suitability in the manufacturing of lightweight aggregate. In addition, it showed that additional additives will improve the properties of lightweight aggregate in terms of specific gravity, strength, and water absorption. Furthermore, the use of geopolymer in lightweight aggregate has been shown to increase the porosity and strength of the aggregate, making it a viable option for lightweight aggregate manufacturing. According to Table 1, various methods can be used to manufacture lightweight aggregate, which are sintering, cold bonding, and autoclaving, which have been reported previously. The sintering and cold bonding methods are the methods that have been used wisely due to the excellent properties of aggregate. Despite the fact that the sintering technique consumes a lot of energy, the quality of the lightweight aggregate generated is excellent, with good strength at a low density. Because of the considerable energy required in the sintering procedure, cold bonding has grown popular because it does not require any additional sintering or heating. This process can create various grade aggregates, depending on the density of the aggregate produced. Generally, aggregate with high density will have a high strength. As a result, the use of a foaming agent may be necessary to make the aggregate lighter. There is relatively limited research on autoclaving methods in the current literature, necessitating more inquiry to assess the possible application of autoclaving methods in the manufacturing of lightweight aggregate. The strength and water absorption capabilities of lightweight aggregate generated by the autoclaving technique were good, and the incorporation of geopolymer improved the properties. As a result, greater research into autoclaving procedures is needed, particularly in terms of simplifying the process so that it can be commercialized.

### 4.1. Sintering

Sintering is a process that consumes high energy to produce artificial aggregate with enhanced properties. As reported by Sun et al. (2021) [34], raw materials with a high amount of SiO_2_ and Al_2_O_3_ commonly use sintering. When the pellets in the disc pelletizer are shaped, the pellets will dry for a day before undergoing the sintering process at a temperature of between 1180 °C and 1200 °C [42,43]. In some cases, some of the pellets are fused at a temperature above 1200 °C for optimum properties [21,44]. Most of the previous research used a similar drying method prior to sintering [45,46]. Chen et al. [47] also reported a similar method where the pellets undergo a drying stage, followed by preheating at 500 °C expanding temperature of a temperature between 1100 °C and 1150 °C for the sintering process.

Meanwhile, according to Grygo and Pranevich [48], the aggregate produced through the sintering process is lighter and has high strength performance. The sintering process is a popular application for mass manufacture of lightweight aggregates that does not require a long-term curing process [49]. Lytag, Pollytag, LECA, and liapour are some of the commercially sintered lightweight aggregates around the world. The factory that manufactured LECA has three lightweight aggregate production lines with a total capacity of 750,000 cubic meters per year [50]. Sintered artificial lightweight aggregate is one of the possible materials to make concrete lighter than the standard aggregate concrete [5]. In addition, Tian et al. (2021) [51] state that sintering aggregates with the help of geopolymerization reactions can have higher aggregate strength and low density. However, the sintering process involves a high level of energy during pelletization, which results in higher manufacturing costs [44]. Aside from that, the sintering process generates a large amount of pollutants, which will cause environmental problems. The sintering method needed a lot of energy regardless of its potential engineering properties with respective mix design applied [52].

In short, the lightweight aggregate produced at the temperature of 1200 °C provides the best properties of the aggregate. To acquire the best features of lightweight aggregate, the suggested sintering temperature for metakaolin is 900 °C, 1100 °C for sewage sludge and river sediment, and 900 °C for fly ash. As a result, materials such as metakaolin and fly ash are more advantageous due to the energy savings at the lowest sintering temperature required to manufacture lightweight aggregate. The sintering method will also be able to produce lightweight aggregate in a shorter time, at which it is suitable to be used to replace natural aggregate. Nevertheless, the sintering method will require high energy during the production, and this will increase the price of the production. The usage of sintered lightweight aggregate in the construction field will increase the overall cost of construction. As a result, new approaches, such as cold bonding, are being studied to address the problems that the sintering method encountered.

### 4.2. Cold Bonding

Cold bonding is a process of enhancing fine particles, either by pressure or non-pressure agglomeration methods, forming larger particles. In the cold bonding process, cement or alkaline activator will be chosen as the binder. The cold bonding method has been noted as a cost-effective method as it agglomerates at room temperature [47]. Furthermore, the cold bonding method tends to minimize energy usage when compared to other production processes [21]. For cold bonding, the pellets will be dried at room temperature for 24 h once the shape of the pellets is formed. The pellets are then sealed in the bag until the testing day [22,35,43,47,53,54]. According to Jiang et al. [55], normal curing at room temperature was required for cold-bonded artificial aggregate to achieve the strength. However, aggregates produced using the cold bonding method required curing at room temperature or in an enclosed chamber with steam until the required strength was attained [56]. The major challenge for cold-bonded aggregate is the requirement for a longer hardening period, as it is normally required to cure for 28 days before being discharged and used as construction materials [34].

From both economic and environmental viewpoints, the cold bonding process is fulfilling, as it involves low energy consumption. Wastewater treatment sludge, ground granulated blast furnace slag, rice husk ash, and fly ash are some of the common materials used to produce cold bonding lightweight aggregate. In addition, lightweight aggregate produced by using cold bonding instead of sintering is considered to have a strong effect on customer acceptance, as it reduces the environmental impact [22].

Meanwhile, the addition of nano SiO_2_ from 0.5% to 1.5% during the production of artificial lightweight aggregate leads to increasing water absorption from 12.5% to 30.1% [57]. In addition, the utilization of foaming agents in lightweight aggregate causes more pores and makes the aggregate lighter than cold-bonded artificial lightweight aggregate. This was supported by the high water absorption ranging from 28.7% to 33.5% when compared to cold-bonded artificial lightweight aggregate, which had a water absorption ranging from 15.1% to 18.9% [58].

In a nutshell, the cold bonding method is considered a low-cost method, as it can be hardened at room temperature. Cold bonding has been recognized as a major step forward in the production of lightweight aggregate. Moreover, the cold bonding method is more likely to be adopted by society, as it does not require additional energy during the process. However, in order to improve the properties of the cold-bonded lightweight aggregate, it needs considerable treatment, particularly the use of a foaming agent during the manufacturing process. Another challenge is the curing day for cold bonding lightweight aggregate, which should be reduced to achieve acceptance in the construction industry.

### 4.3. Autoclaving

Autoclaving is a process that involves the addition of chemicals, such as lime or gypsum, in the agglomeration stage. In addition, autoclaving produced aggregates with little binding material and low curing time [21]. For autoclaving, the pellets will be hardened by the autoclave pressure and temperature to gain strength. A previous study by Wan et al. [38] reported autoclaving for the production of aggregates. The quartz tailing aggregate was cured at room temperature for 24 h, followed by curing at a temperature of up to 195 °C for 3 h with an autoclaved pressure of 1.38 MPa. The aggregates were further cured at 195 °C for another 10 h without autoclaved pressure before cooling at room temperature. The cured aggregate was then dried in order to achieve the desired weight of less than 1100 kg/m^3^. The autoclaving method produced aggregates very quickly and it required little binding material and curing time [21]. Moreover, lightweight aggregate can be made with a considerable number of industrial solid wastes utilizing autoclave technology, which not only reduces the curing time (to only 4 h) to maximize space utilization, but also meets commercial environmental and economic requirements [37].

However, there are still limited studies available on autoclaving. This is because the autoclaving method requires an autoclaved machine with the required temperature and pressure to harden the aggregate. In addition, the autoclaved machine is very expensive and requires high power consumption and large production facilities to complete the process.

Generally, the sintering method has been widely used around the world with some popular commercial products, such as LECA and Lytag. LECA is one of the most popular artificial lightweight aggregates that has been commercialized in the market used to replace natural aggregate. The production rate can be up to 200,000 m^3^ per year, depending on local LECA requirements and capital available. LECA is a new revolutionary material, and its manufacturing rate compared to standard aggregate is still dependent on customer demand. However, due to the requirement of high sintering temperature to produce sintered aggregate, the cold bonding method has been introduced as an initiative towards saving energy. The lightweight aggregate produced through the cold bonding method has the potential to be applied in concrete production due to its comparable properties to other methods. Moreover, cold bonding also showed promising properties, such as high compressive strength when applied to the concrete. Cold bonding also contributes to low pollutant production and low operating expenses. In addition, the autoclaving method is also another method that can be used to produce artificial lightweight aggregate. However, an autoclaved pressure machine is required for this method, which is very costly. The autoclaving method also required longer curing time to achieve the strength of the aggregate. Therefore, among all of the commonly reported methods, the cold bonding method with proper optimization of mix design is noted to be advantageous to the construction field. Furthermore, variations in manufacturing methods, as well as mix designation, were known to have a significant impact on the properties of lightweight aggregate, particularly on physical and mechanical properties.

## 5. Physical and Mechanical Properties of Lightweight Aggregate

### 5.1. Specific Gravity

The specific gravity of the aggregate varies depending on the type of raw material used. The cold-bonded fly ash aggregate that used different molarities of alkaline activator had a specific gravity of between 1.8 and 1.85 [22]. In addition, mixing 90% of fly ash with 10% of cement by using cold bonding gives a specific gravity of 1.76 [56]. The artificial aggregate that was made up of fly ash by using the cold bonding method had a specific gravity of 1.63 as compared to normal coarse aggregate at 2.71 [54]. The specific gravity was found to be increased from 1.84 to 1.91 when the styrene–butadiene rubber (SBR) was added from 1% to 3% to the lightweight aggregate [35]. In addition, the aggregate produced by mixing bentonite and metakaolin together with fly ash has a specific gravity of 1.8 to 1.93 and 1.95 to 1.99 [59].

The sintered fly ash aggregate had a specific gravity between 1.41 and 1.44, with a size that varied from 2 mm to 12 mm [5]. The specific gravity of aggregate that was made from water treatment residual increased from 1.21 to 1.78 when the sintering temperature was increased from 1000 °C to 1100 °C [42]. The sintered dredged sludge lightweight aggregate had a specific gravity of 1.00 to 1.38 for the particle size of 4.75 mm to 12.5 mm [47]. The sintered fly ash aggregate with bentonite added had a specific gravity of 1.57, while the sintered fly ash aggregate with glass powder added had a specific gravity of 1.60 at the temperature at 1200 °C [54]. Meanwhile, coarse aggregate manufactured from bentonite and water glass has a specific gravity of 1.63 at a temperature of 800 °C [46].

In comparison to natural aggregates, the specific gravity of artificial geopolymer aggregates formed by sintering is quite low [21]. For instance, Kamal and Mishra (2020) [60] reported on the specific gravity of the fly ash aggregates as well as raw materials, including fly ash and binder, and the amount of void space in the aggregate. In addition, whenever cold-bonded aggregate was combined with other pozzolanic binding materials, such as GGBS, the specific gravity was found to be as high as 2.42, in which the hydrated lime acts as a primary binder [61]. Previous research on the determination of specific gravity of aggregates can be summarized as in Table 2.

From Table 2, the specific gravity of lightweight aggregate was found to be in the range from 1.41 to 2.2. Based on BS EN 13055-1 [17], the specific gravity of lightweight aggregate should be less than 2.0. The wide range of the specific gravity of lightweight aggregate values can be explained by the influence factors, including type of material used, type of method used, and type of binder used during the manufacturing process. The specific gravity of lightweight aggregate is often increased by adding additives. Furthermore, the specific gravity of lightweight aggregate is affected by the curing temperature, with a greater curing temperature resulting in a lower specific gravity. The addition of a foaming agent will aid in lowering the specific gravity. In addition, the sintering method always was proven to have lower specific gravity compared to other methods due to the formation of voids at higher temperatures. However, the low specific gravity can be achieved by the cold bonding and autoclaving methods through addition of additive, such as protein-based foaming agent.

### 5.2. Water Absorption

Water absorption provides an indication of the internal aggregate structure. Higher water absorption of aggregates indicates the large number of pores in nature and usually gives drawbacks to the aggregates. For instance, expanded perlite powder (EPP) was noted as being high in porosity, thus causing the water absorption to increase from 33% to 52% when the replacement of fly ash increased to 30% [53]. Meanwhile, when sintering is applied, increasing the sintering temperature was found to decrease water absorption of all aggregates due to the increment in the fusion of material, which led to less water surface permeability [48]. In the study conducted by Sun et al. (2021) [65], it was found that the sintered aggregate made up of red mud and municipal solid waste incineration bottom ash with the temperature increased from 1010 °C to 1090 °C caused the porosity of the aggregate to increase and the water absorption to decrease significantly until 1070 °C. Furthermore, Liu et al. (2018) [66] also found that lower water absorption can be obtained when the lightweight aggregate was sintered at a temperature of around 1100 °C. Lightweight aggregate made up of drill cuttings containing synthetic-based mud, when sintered at 1180 °C, had water absorption of 3.6% [24]. In addition, the water absorption of metakaolin artificial lightweight aggregate increases at the sintering temperature over 900 °C. The pores formed during the sintering process were found to be closed pores, thus causing a reduction in permeability to water [67].

In addition, the addition of the waste glass powder to lightweight aggregates was found to significantly reduce water absorption from 7.73% to 0.5% [30]. Meanwhile, due to the porosity of the hydrated calcium silicate, the quartz tailing aggregate (QTA) also possessed high water absorption, which varies from 13.77% to 21.93% [38]. In another study, the water absorption was reduced from 12.1% to 8.58% when styrene–butadiene rubber (SBR) was added from 1% to 3% to the lightweight aggregate, which proved the minimizing of voids when the SBR increased in the pellets [35]. Furthermore, it was found that the lightweight aggregate produced from different ratio palm oil fuel ash and silt causes high water absorption of 32.2% when 90% of clay is used [8]. This is due to the high pozzolanic reaction rate within the mixture, thus causing higher water absorption through the capillary of silt. Moreover, water absorption for aggregates incorporated with 10% cement was lowered by 13.97% due to a stronger hydration reaction and a denser microstructure with more C-S-H and CH products, thus leading to an increase in the strength of the aggregate [68].

In addition, the utilization of alkaline activator as a binder for the production of lightweight aggregate was found to increase the water absorption from 22% to 23% [22]. Meanwhile, the geopolymer lightweight aggregate sintered using microwave radiation had water absorption of 18.98%, as it was affected by the high density of the aggregate [29]. In another study, it was found that the pores in the fly ash geopolymer aggregate were reduced after the geopolymerization process, thus giving a denser microstructure, which resulted in lower water absorption at 10.05% [32]. Meanwhile, increasing the Na_2_SiO_3_/NaOH ratio from 1.5 to 4.0 caused the water absorption values for geopolymer lightweight aggregate to steadily increase from 15.2% to 19% due to the foaming activity of Na_2_SiO_3_ [58]. Furthermore, the metakaolin geopolymer aggregate sintered at 600 °C will have lower water absorption as the greater the sintering temperature, the more voids created, and, hence, the higher the water absorption of lightweight aggregate [69]. Moreover, the fly ash geopolymer aggregates had higher water absorption when curing at 80 °C due to the water in the aggregates participating in the geopolymerization process, which improves the strength of the pellets [64].

From Table 3, the water absorption for artificial lightweight aggregate was proven to be higher than that of the natural aggregate, which can be explained by the effect of porosity due to pore formation. In addition, the water absorption of the lightweight aggregate can be affected by influence factors, including the type of materials used, the type of curing, and the type of binder used. The sintering method demonstrated that, when the temperature rises, the water absorption of lightweight aggregate decreases because it contains closed pores. The majority of research has indicated that the cold bonding procedure will have higher water absorption and will require additional treatment to eliminate this problem. Furthermore, adding additives to the lightweight aggregate will aid in water absorption reduction. The addition of geopolymer to lightweight aggregate will increase water absorption significantly. Water absorption will be reduced by the presence of a vitrified shell around the artificial lightweight aggregate. As a result, the water absorption of lightweight aggregate has an impact on mechanical qualities and should be assessed before using it in concrete.

### 5.3. Mechanical Properties

In terms of mechanical properties, fly ash with expanded perlite powder (EPP) has a higher crushing strength due to increasing pozzolanic activity [53]. Sintered sediment lightweight aggregate with a bulk density of 859 kg/m^3^ had the highest crushing strength of 13.4 MPa. This occurrence proves that the aggregate strength increases with increasing bulk density [47]. The crushing strength decreased as the silt content increased due to the binding failure of palm oil fuel ash (POFA) with the silt content [8]. Meanwhile, the fly-ash metakaolin binder aggregate showed high crushing strength when curing under high temperatures [59]. For instance, when the sintering temperature exceeds 900 °C, the sintered aggregate made up of metakaolin and alkaline activator has high aggregate impact value, which cause decreasing aggregate strength due to the increasing amounts of pore space in the aggregate [67]. Moreover, combination of fly ash and clay with 10% of sodium carbonate and sintering temperature of 1220 °C leads to a higher pellet strength of 4.25 MPa [31].

In addition, regardless of methods applied, the cold-bonded fly ash aggregate, sintered fly ash aggregate, as well as autoclaved aggregates were found to have a high impact value of 9.56%, 10.2%, and 11.46%, respectively [71]. According to research of Kamal and Mishra (2020) [60], the addition of binder is noted as effective due to the binder’s role, which is to wrap the pellets, therefore causing the voids to have better resistance to compression. Meanwhile, the addition of styrene–butadiene rubber (SBR) to lightweight aggregate leads to a lower impact value, which makes the aggregate stronger [35]. The impact resistance of cement-based fly ash aggregate was enhanced by the cement content because of the increased hydration reaction. In addition, the curing temperature will also increase the impact resistance of artificial aggregate [33]. The high porosity of phosphogypsum-based cold-bonded aggregates with 90% of phosphogypsum accounts for high water absorption with 13.6%, as it holds fewer binders and allows it to absorb more water [72]. The inclusion of cement enhanced the pellet strength from 1 MPa to 2.3 MPa when compared to the pellet strength of the cold-bonded lightweight aggregate made with only concrete slurry waste and fine incinerator bottom ash [36].

In addition, the lightweight aggregate with the lowest water absorption has better sustainability towards the impact of the load. In a previous study, it was discovered that increasing the maximum amount of fly ash replacement with 10% cement or 5% calcium hydroxide increased cold-bonded aggregate strength with decreasing water absorption [70]. On the other hand, curing at higher temperatures causes the impact value of artificial lightweight aggregate to improve by 12.5% to 14.75% and the crushing strength by 28.2% to 39.7% [64]. According to a study by Rehman et al. (2020) [68], the aggregate with the lowest water absorption of 12.5% had the lowest aggregate impact value of 22.12%, thus proving the stronger microstructure and lower porosity had led to high resistance to crack penetration and increasing strength. Meanwhile, according to Ghosh (2018) [73], the autoclaved aggregate made by using fly ash and cement can be used to replace the gravel as the crushing value and impact value due to the similar value.

From the previous studies in Table 4, it is shown that the mechanical properties of lightweight aggregate mainly depend on the type of material used. Furthermore, adding additives to the lightweight aggregate can aid to improve its strength. The majority of the researchers concluded that adding geopolymer to the aggregate leads to an improvement in strength performance. The method of curing, on the other hand, will have an impact on the strength of the lightweight aggregate, as a higher curing temperature will result in greater strength. The mechanical properties of the lightweight aggregate can be affected by the microstructure of the lightweight aggregate.

### 5.4. Morphology

**Figure 2:**
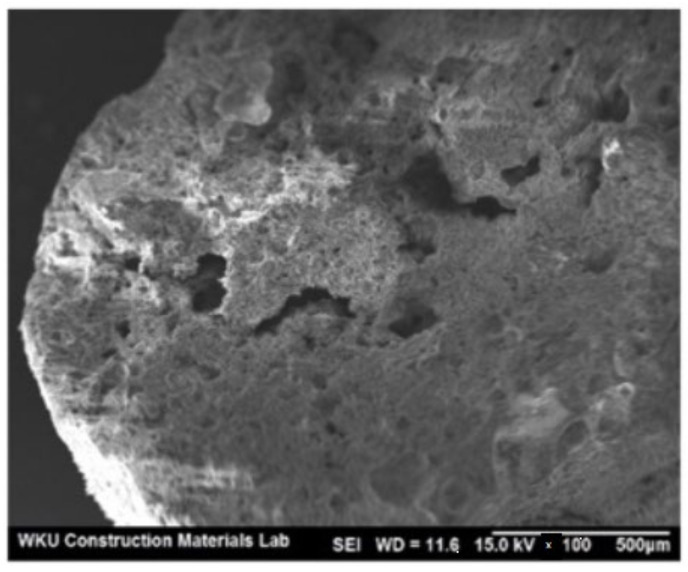
SEM of artificial lightweight aggregate made up of calcining coal ash and dredged soil [77].

The morphology of lightweight aggregate can be observed through scanning electron microscopy (SEM). The artificial lightweight aggregate that was made from calcining coal ash and dredged soil was observed through SEM, and the morphology can be shown as in Figure 2. The observation of voids from the morphology proved that the formation of voids contributes to lower specific density and loose bulk density.

**Figure 3:**
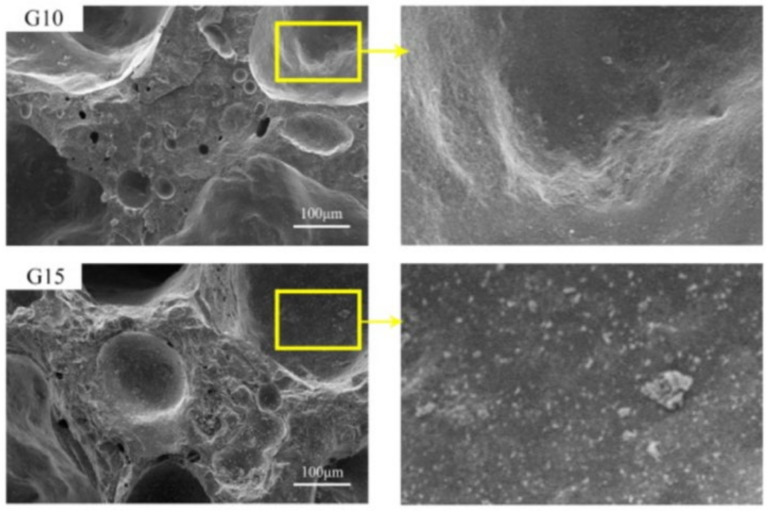
SEM of sintered lightweight aggregate samples with 10% and 15% of water glass content at 1180 °C [30].

**Figure 4:**
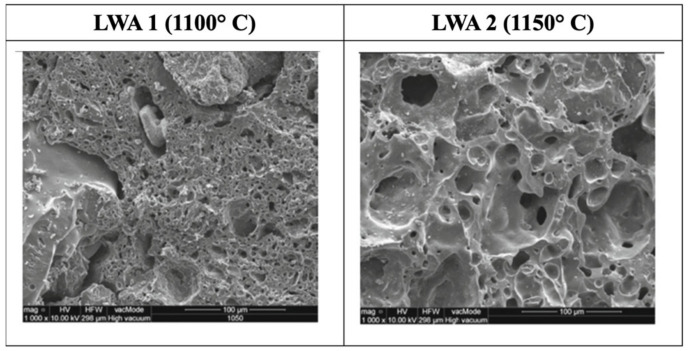
SEM of sintered lightweight aggregate made up of clay and sewage sludge at 1100 °C and 1150 °C [18].

Figure 3 shows SEM images of sintered lightweight aggregate at 1180 °C. The increasing amount of waste glass powder in the sample allows the tiny voids to be filled up, thus causing less porosity of the sintered lightweight aggregate with increasing particle density [30]. Besides that, excess gas may be created when using the sintering method. The formation of pores will occur continuously when the temperature applied is too high [78]. In addition, for the sintering method, the lightweight aggregate sintered at 1100 °C had a smooth and thick surface with solitary and round pores with widths ranging from 10 to 20 μm which minimizes porosity. This will lead to enhanced densification by creating samples with minimal water absorption and high compressive strength [66]. As shown in Figure 4, the pores in the aggregates LWA1 and LWA2 are significantly larger because organic substance components derived from sewage sludge release gases that aid in the development of pores and thus form a porous aggregate structure [18].

**Figure 5:**
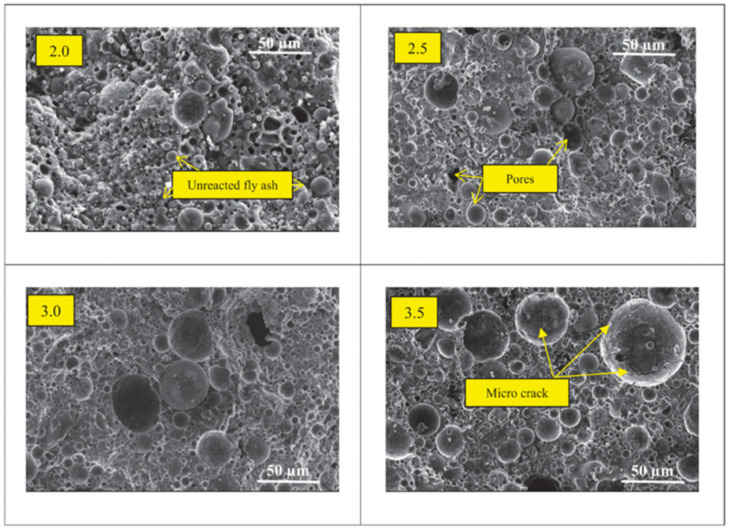
SEM of geopolymer aggregate at solid/liquid ratio of 2.0, 2.5, 3.0, and 3.5 [76].

**Figure 6:**
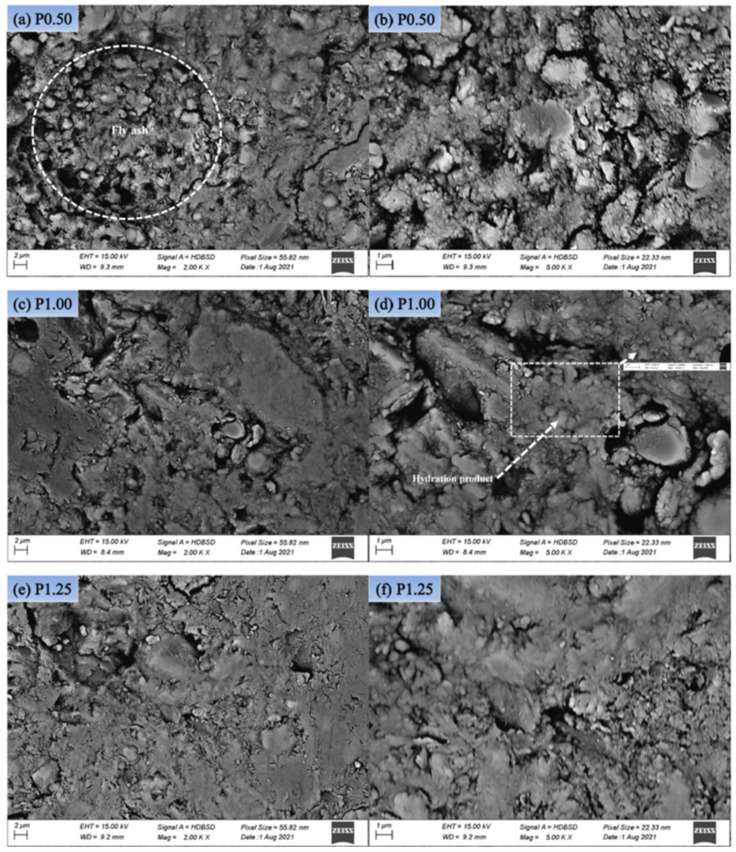
SEM of autoclaved lightweight aggregate with different curing pressure: (**a**,**b**) P0.50, (**c**,**d**) P1.00, (**e**,**f**) P1.25 [37].

In addition, alkaline activators such as sodium hydroxide and sodium silicate have been used previously as liquid precursors and mixed with aluminosilicate materials such as fly ash and rice husk ash to create a cold bonded lightweight aggregate known as geopolymer aggregate, and the result can be depicted as in Figure 5. The denser matrix that is shown in Figure 5 for geopolymer aggregate with the solid-to-liquid (fly ash/alkaline activator) ratio of 3.0 results in a lower AIV value, where the strength of the geopolymer aggregate is higher. The geopolymer aggregates indicated that excellent solidification of the fly ash with alkaline activators occurred by geopolymerization reaction as the alkaline activator dissolved most of the fly ash particles [79]. Furthermore, the SEM of quarry tailings autoclaved aggregate in Figure 6 showed that high stream curing had a denser structure, which increased the strength alongside with reduced water absorption of the aggregate [37].

From the morphology of lightweight aggregate, the pore of the lightweight aggregate can be observed through SEM. The formation of pores observed from the morphology is significant towards proving the increment and decrement in some properties of aggregates, such as specific gravity and density. Based on the previous study, aggregate that was made up of fly ash through geopolymerization showed the highest distribution of pores. The connected pores lead to higher water absorption than disconnected pores due to the ability to absorb more water into the aggregate. Meanwhile, the denser structure that was observed through SEM can provide good properties of lightweight aggregate, which can bring benefits in the application of concrete.

## 6. Lightweight Aggregate Concrete

### 6.1. Mechanical Properties

The utilization of lightweight aggregate also affects the significant properties of the concrete produced, including mechanical properties, thermal conductivity, and ultrasonic pulse velocity. The mechanical properties of lightweight aggregate concrete determine the suitability of artificial lightweight aggregate used in the concrete. The interfacial zone (ITZ) between the coarse aggregate and paste is a critical factor that will affect the mechanical properties of the concrete [80].

Compressive strength is the ability of the structure to resist compression, and this is the main design variable for engineers. The compressive strength of mortar containing fly ash aggregate will increase slowly at the beginning of the hardening stage, but it will increase rapidly after 14 days. In addition, the heavier the mortar, the higher the compressive strength. For instance, mortar of fly ash with an 8 M concentration of NaOH shows the heaviest bulk density with the highest compressive strength [22]. The compressive strength for quartz tailings aggregate (QTA) concrete reaches 74 MPa, which is considered high strength concrete. This is because the cement binders are readily penetrated to form a later mechanical interlocking structure around the aggregates to strengthen the bonding between aggregate and cement paste since the shell of QTA is a porous, fibrous, and needle-flake tobermorite structure [38]. The increasing amount of styrene-butadiene rubber (SBR) in pellets will cause an increase in the compressive strength of SBR modified lightweight aggregate (SLWA) concrete. This is due to the microstructure of the SLWA, which generates a strong bond between the aggregate and the cement paste [35]. Due to the impact on the restriction of the spread of cracking occurring, the beneficial effect of the addition of fibres to the lightweight aggregate cement mix gives a higher compressive strength (over 40 MPa) and can be used as a structural application in the construction field [81]. Table 5 shows the compressive strength of lightweight aggregate concrete reported by past researches. According to Table 5, it can be concluded that the artificial lightweight aggregate concrete had achieved the compressive strength for structure concrete which is 17 MPa based on ACI 318M-14.

In general, the concrete that used the autoclaved quartz tailing lightweight aggregate had a compressive strength of up to 74 MPa and alkali-activated fly-ash-based artificial lightweight aggregate achieved 64 MPa of compressive strength at 28 days. In addition, when lightweight aggregate has been used in concrete, it achieved early strength compared to conventional concrete. The additives added to the lightweight aggregate enhanced the aggregate’s strength, which will also increase the lightweight concrete’s strength. The use of lightweight aggregate in concrete met the minimal compressive strength requirements for the use of structural components. As a result, artificial lightweight aggregate has been an innovative material that can be used to manufacture lightweight structural components that have been highlighted in the recent construction field to minimize the structure’s dead load and protect it from earthquakes.

### 6.2. Thermal Conductivity

**Figure 7:**
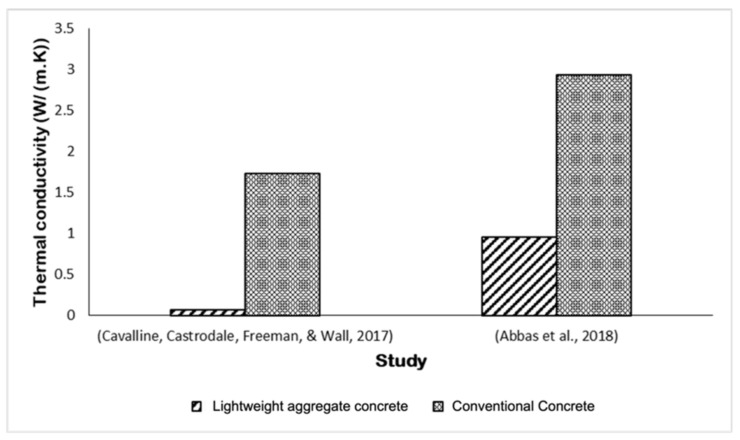
Previous studies’ comparison on thermal conductivity between lightweight aggregate concrete and conventional concrete.

The capacity of a substance to transport heat is measured by thermal conductivity, which is significant in determining the insulation of materials. Lightweight concrete normally has low thermal conductivity compared to conventional concrete [3]. The thermal conductivity of lightweight concrete is 0.9567 W/m·K, whereas the thermal conductivity of normal weight aggregate is 1.98–2.94 W/m·K [46]. The low thermal conductivity of lightweight concrete can be due to the type of material used to produce lightweight aggregate. According to Tajra et al. [53], the low conductivity of lightweight concrete is due to the use of expanded perlite as the core structure, which has a low thermal conductivity of about 0.05 W/m·K, as well as the use of expanded perlite powder in the shell structure, which improved its thermal properties. Concrete with lightweight coarse and fine aggregate has the lowest thermal properties, 0.0703 W/m·K, when compared to conventional concrete, which has thermal properties of 1.736 W/m·K [2]. Figure 7 shows the comparison between lightweight aggregate concrete and conventional concrete. Based on Figure 7, the lightweight aggregate concrete had decreased by 95.95% and 67.46% as compared to normal aggregate concrete. The application of sintered expanded slate aggregate in coarse and fine aggregate showed that it had the lowest thermal conductivity. Therefore, it can be concluded that the application of sintered lightweight aggregate in concrete is more suitable to be used as thermal insulation material. It would be fascinating to look into finding the thermal conductivity of concrete using cold-bonded lightweight aggregate and autoclaved lightweight aggregate.

### 6.3. Ultrasonic Pulse Velocity

Ultrasonic pulse velocity (UPV) testing is used to verify the integrity and quality of structural concrete alongside voiding, honeycombing, cracking, and other defects. According to BS 1881-203 [83], the ultrasonic pulse velocity, which is greater or equal to 4.5 km/s, is considered as excellent concrete quality, while less than 2.0 km/s is classified as very weak concrete quality. Based on the study conducted by Tanaka et al. (2020) [4], the pulse velocity is 3.5 km/s to 4.4 km/s, which is slower as compared to the control sample due to the large amount of air voids in the artificial aggregate in the concrete, which affects the quality of the concrete. In study of Rehman et al. (2020) [68], it was found that geopolymer-based lightweight concrete has a higher ultrasonic pulse velocity, which is 2936 m/s to 3016 m/s as compared to cement-based lightweight concrete, which is 2601 m/s to 2835 m/s. This can be explained by the higher strength and more complex microstructure of the geopolymer-based concrete. Further, the occurrence of large amounts of micro-cracks in the concrete can cause the ultrasonic pulse velocity to be lowered, which is 3.42–4.51 km/s when the replacement of coarse and fine aggregates is increased with artificial lightweight aggregate (Satpathy et al., 2019) [63]. For geopolymer concrete specimens containing artificial lightweight aggregate, the ultrasonic pulse velocity value vary from 4.15 km/s to 4.35 km/s, which can be considered good quality concrete (Abbas et al., 2018) [46]. Furthermore, the UPV of the concrete mixes decreased from 4.29 km/s to 3.58 km/s as the lightweight expanded clay aggregate (LECA) and expanded perlite aggregate (EPA) replacement percentage increased, and this could be due to the existence of voids in LECA and EPA lengthening the travel path of the ultrasonic pulse and resulting in a lower UPV value [84].

From Table 6, it can be concluded that the lightweight aggregate concrete will provide good ultrasonic pulse velocity to prevent the defects of the concrete. It is also possible to deduce that the majority of the ultrasonic pulse velocity of concrete is determined by the use of commercialized lightweight aggregate, such as LECA, which is manufactured using the sintering method. As a result, the ultrasonic pulse velocity for concrete with cold-bonded lightweight aggregate and autoclaved lightweight aggregate is limited, and more research is needed.

## 7. Conclusions

Based on the review, the cold bonding method and the autoclaving method can be considered as alternative ways to produce the lightweight aggregate due to the comparable properties to the sintering method. However, the aggregate produced from the cold bonding method and the autoclaving method are still limited and require further exploration in order to be commercialized as a sintering method. Other than features such as water absorption and specific gravity, the curing days for aggregate generated should be researched further for the cold bonding process, as this approach requires the same curing days as OPC. The construction sector will accept shorter curing days since they are more practical to commercialize. The performance of the aggregate produced by the autoclave process is good; however, the low temperature during autoclave pressure may need to be researched more in the future to lower production costs and, thus, make it a viable alternative to commercially available good-grade aggregate.

Further, lightweight aggregate having a density of less than 2.0 can be utilized extensively in lightweight concrete to lower the structure’s dead load. The specific gravity of lightweight aggregate will be mostly affected by the type of material and binder used. In addition, the artificial lightweight aggregate showed lower water absorption than normal aggregate. The water absorption of lightweight aggregate is increased when geopolymer is used in the manufacturing process, and it can be further improved by utilizing additional treatments, such as vacuum impregnation or coating. Furthermore, due to the fact that the pozzolanic activity with the inclusion of geopolymer in the lightweight aggregate is higher, mechanical properties of lightweight aggregate are improvable. Moreover, the morphology of lightweight aggregate can help to determine the microstructure of lightweight aggregate. Lightweight aggregate with a denser microstructure and lower porosity was found to be more resistant to crack penetration and have higher strength. The properties of lightweight aggregate can be improved by a greater distribution of internal aggregate holes and fewer continuous pores observed on the microstructure.

On the other hand, the interfacial zone (ITZ) between coarse aggregate and paste is a significant factor that will affect the compressive strength of the concrete. Thus, more research into the bonding mechanism between aggregate and cement matrix is critical. The compressive strength of lightweight aggregate concrete can be increased by adding additives, such as synthetic fibers. Higher compressive strength can be obtained by using lightweight aggregate in order to construct lightweight structural components to protect it from earthquake resistance. Additionally, lightweight aggregate with geopolymer demonstrates early strength development in lightweight concrete. The low thermal conductivity of lightweight aggregate concrete can be applied in the construction field as a thermal insulation material. This can assist the building to be more comfortable while using less energy. Furthermore, the application of lightweight aggregate in the concrete will produce excellent quality structural concrete, which can reduce defects when applied in the construction field. Therefore, the creation of lightweight aggregate can be a unique material that can be used in a variety of applications to minimize the usage of natural aggregate and bring benefits to the environment, such as reducing the dead load of the structure, enhancing thermal insulation properties, and reducing CO_2_ emissions.

## Figures and Tables

**Table 1:** Previous studies on the type of method to manufacture lightweight aggregate.

Researcher	Method	Raw Materials	Additives	Significant Finding
Kwek et al. (2022) [16]	Sintering	Palm Oil Fuel Ash and Silt	Alkaline activator (NaOH and Na_2_SiO_3_) and lime	- The bulk density can obtain as low as 1.18 kg/m^3^ - The individual crushing strength is almost the same as commercialized aggregate used in lightweight concrete
Kwek and Awang (2021) [23]	Sintering	Palm Oil Fuel Ash	Alkaline activator (NaOH and Na_2_SiO_3_) and lime	- The strength of the aggregate achieved to be utilized for lightweight constructions or insulating concrete
Li et al. (2020) [30]	Sintering	Sewage sludge	Waste glass powder	- The addition of waste glass powder helps in reduction of water absorption
Ren et al. (2020) [31]	Sintering	Fly ash and clay	Coke particles	- Coke particles reduce the apparent density
Chien et al. (2020) [19]	Sintering	Industrial sludge and marine clay	Na_2_CO_3_	- The Na_2_CO_3_ can reduce the specific gravity and the firing temperature required for production of lightweight aggregate
Abdullah et al. (2021) [32]	Cold bonding	Fly ash	Alkaline activator (NaOH and Na_2_SiO_3_)	- NaOH molarity will affect the strength of the aggregate - 12 M of NaOH provide the optimum mix design of geopolymer aggregate
Risdanareni et al. (2020) [22]	Cold bonding	Fly ash	Sodium hydroxide (NaOH) solution	- 6 M of NaOH brought a positive impact to aggregate strength - The highest compressive strength at 8 M of NaOH
UI Rehman et al. (2020) [33]	Cold bonding	Fly ash, Slag	Cement (Cement based) and Alkaline activator (Geopolymer based)	- Lightweight aggregate produced from cement based is strongest - Aggregate with hot water curing shows good properties as it can withstand half of the compressive load as compared to normal aggregate
Vali and Murugan (2020) [34]	Cold bonding	Fly ash, GGBS, hydrated lime	glass fibers	- Lightweight aggregate produced achieved the requirement for structural components
Patel et al. (2019) [35]	Cold bonding	Fly ash	Styrene-butadiene rubber	- Compressive strength increase compared to normal lightweight aggregate concrete
Tang et al. (2019) [36]	Cold bonding	Concrete slurry waste (CSW) and fine incineration bottom ash (IBA)	Cement and ground granulated blast furnace slag (GGBS)	- Addition of cement or GGBS as the additives in the manufacturing process will increase the strength of the aggregate
Wang et al. (2022) [37]	Autoclaved	Quartz tailings, fly ash, cement	Alkaline activator (NaOH and Na_2_SiO_3_)	- The strength of aggregate increase from 7.61 MPa to 10.20 MPa when increasing the autoclaved pressure - The water absorption decreases from 2.22% to 1.83% when increasing the autoclaved pressure
Wang et al. (2020) [38]	Autoclaved	Quartz tailings, fly ash	Quicklime	- The compressive strength is high - Higher structure efficiency for quartz tailing aggregate concrete

**Table 2:** Previous studies on specific gravity of lightweight aggregate.

Researcher	Aggregate	Specific Gravity
Shahane and Patel (2021) [62]	Cold-bonded Fly ash aggregate at 75 °C	2.1
	Cold-bonded Fly ash aggregate at 65 °C	2.2
Risdanareni et al. (2020) [22]	Cold-bonded Fly ash-based artificial lightweight aggregate	1.8–1.85
Kamal and Mishra (2020) [60]	Sintered Fly ash aggregate	1.52–1.9
	Sintered Fly ash aggregate (bentonite as binder)	1.61–1.65
	Sintered Fly ash aggregate (glass powder as binder)	1.64–1.67
Satpathy et al. (2019) [63]	Sintered fly ash lightweight aggregate	1.89
Nadesan and Dinakar (2018) [5]	Sintered fly ash lightweight aggregate	1.41–1.44
Abbas et al. (2018) [46]	Sintered lightweight aggregate produced from bentonite clay and water glass (sodium silicate)	1.63
Shivaprasad and Das (2018) [64]	Cold-bonded fly ash aggregate (heat cured)	1.94–2.03

**Table 3:** Previous studies on water absorption of lightweight aggregate.

Researcher	Aggregate	Water Absorption (%)
Ren et al. (2020) [31]	Artificial aggregate (fly ash + clay)	8.7
	Artificial aggregate (fly ash + clay + coke particles)	22.97
	Artificial aggregate (fly ash + clay + sodium carbonate solution)	8.84
Risdanareni et al. (2020) [22]	Alkali activated Fly ash-based artificial lightweight aggregate (4 M NaOH)	23.92
	Alkali activated Fly ash-based artificial lightweight aggregate (6 M NaOH)	23.23
	Alkali activated Fly ash-based artificial lightweight aggregate (8 M NaOH)	22.08
Rehman et al. (2020) [68]	Lightweight aggregate (fly ash + GGBS + 10% cement)	14.53
	Lightweight aggregate (fly ash + GGBS + 20% cement)	12.50
Vali and Murugan (2019) [57]	Cold-bonded artificial aggregate (fly ash + hydrated lime + cement + nano SiO_2_)	22.9–30.1
	Cold-bonded artificial aggregate (fly ash + hydrated lime + metakaolin + nano SiO_2_)	20.7–28.2
	Cold-bonded artificial aggregate (fly ash + hydrated lime + slag + nano SiO_2_)	12.5–23.8
Narattha and Chaipanich (2018) [70]	Cold-bonded fly ash lightweight aggregates	14.08
	Cold-bonded fly ash lightweight aggregates (Additional of Portland cement)	13.34–16.90
	Cold-bonded fly ash lightweight aggregates (Calcium hydroxide)	14.10–18.46
Mohamad Ibrahim et al. (2018) [44]	Cold-bonded lightweight aggregate (cured at room temperature)	22.1–39.8
	Cold-bonded lightweight aggregate (cured under water at room temperature)	21.1–39.0
	Cold-bonded lightweight aggregate (cured at oven)	26.5–41.3
	Cold-bonded lightweight aggregate (cured under water at oven)	24.5–39.5

**Table 4:** Previous studies on mechanical properties of lightweight aggregate.

Researcher	Aggregate	Crushing Strength (MPa)	Aggregate Impact Value, AIV (%)
Ding et al. (2022) [72]	Phosphogypsum-based cold-bonded aggregates	8.11–11.04	-
Zafar et al. (2021) [58]	Foam Lightweight Aggregate	0.35–0.83	-
	Geopolymer Lightweight Aggregate	3.69–4.14	
Aslam et al. (2020) [74]	Geopolymer Lightweight Aggregate (fly ash, silica fume, baking soda)	3.34–4.54	10.03
Saleem et al. 2020 [29]	Geopolymer lightweight aggregates sintered by microwave radiations	3.08–3.96	22.1–35.7
Saad et al. (2019) [75]	Artificial granular lightweight aggregates (bottom ash + cement)	4.0–7.13	25.5–42.5
Taijra et al. (2018) [53]	Core-shell structured lightweight aggregate (expanded perlite powder + fly ash)	2.04–2.66	-
Shivaprasad and Das (2018) [64]	Fly ash aggregate (Ambient Cured)	2.87	27.57
	Fly ash aggregate (60 °C Cured)	3.68	24.10
	Fly ash aggregate (80 °C Cured)	4.01	23.50
Mohamad Ibrahim et al. (2018) [44]	Cold-bonded lightweight aggregate (cured at room temperature)	-	17.2–57.9
	Cold-bonded lightweight aggregate (cured under water at room temperature)		15.4–55.7
	Cold-bonded lightweight aggregate (cured at oven)		25.4–61.0
	Cold-bonded lightweight aggregate (cured under water at oven)		22.1–58.5
Abdullah et al. (2018) [76]	Fly ash geopolymer artificial aggregate (fly ash/alkaline activator = 2.0)	-	23.19
	Fly ash geopolymer artificial aggregate (fly ash/alkaline activator = 2.5)		23.14
	Fly ash geopolymer artificial aggregate (fly ash/alkaline activator = 3.0)		19.6
	Fly ash geopolymer artificial aggregate (fly ash/alkaline activator = 3.5)		25.56

**Table 5:** Previous studies on compressive strength of lightweight aggregate concrete.

Researcher	Aggregate	28 Days of Compressive Strength (MPa)
Risdanareni et al. (2020) [22]	Alkali-activated fly-ash-based artificial lightweight aggregate	64.0
Sahoo et al. (2020) [81]	Sintered fly ash aggregate with synthetic fiber	46.0
Wang et al. (2020) [38]	Autoclaved quartz tailing lightweight aggregate	74.0
Patel et al. (2019) [35]	SBR-modified lightweight aggregate	42.0
Abbas et al. (2018) [46]	Sintered fly ash aggregate	35.8
Lau et al. (2018) [82]	Sintered lime-treated sewage sludge and palm oil fuel ash	50.4

**Table 6:** Previous studies on ultrasonic pulse velocity of lightweight aggregate concrete.

Researcher	Ultrasonic Pulse Velocity	Concrete Quality Based on BS:118-203	Material
Othman et al. (2020) [84]	3.58 km/s to 4.21 km/s	Good	Lightweight Expanded Clay Aggregate (LECA) and Expanded Perlite Aggregate (EPA)
Tanaka et al. (2020) [4]	3.5 km/s to 4.4 km/s	Good	Lightweight artificial aggregate from industrial by-product
Satpathy et al. (2019) [63]	3.42 km/s to 4.51 km/s	Good, 4.51 (Excellent)	Fly ash cenosphere and sintered fly ash aggregate
Abbas et al. (2018) [46]	4.15 km/s to 4.35 km/s	Good	Sintered fly ash aggregate

## Data Availability

Not applicable.
